# Attenuated NER Expressions of XPF and XPC Associated with Smoking Are Involved in the Recurrence of Bladder Cancer

**DOI:** 10.1371/journal.pone.0115224

**Published:** 2014-12-23

**Authors:** Jianhong Qiu, Xiangwei Wang, Xiaodong Meng, Yan Zheng, Gang Li, Jiyao Ma, Gang Ye, Yong Li, Jie Li

**Affiliations:** 1 Department of Urology, Bethune International Peace Hospital, Shijiazhuang, China; 2 Department of Urology, The Second Affiliated Hospital, The Third Military Medical University, Chongqing, China; University of California at Davis, United States of America

## Abstract

The varied NER genes and smoking are two important risk factors of bladder cancer, but the mechanism of the NER protein and smoking in cancer progression, however, remains unclear. In this report, we compared the expressions of NER genes in 79 bladder cancer tissues with or without any recurrence by real-time PCR and then analyzed the varied NER genes by immunochemistry in 219 bladder cancer tissue samples. Based on the clinical data, we analyzed the clinical value of varied NER genes and smoking in 219 bladder cancers by the Kaplan-Meier method and Cox proportional hazards regression. We found the expressions of the NER gene XPF and XPC were significantly lower in bladder cancer tissues with a recurrence compared with those without a recurrence at mRNA level. Also, the patients with the XPF and XPC defect had a statistically significant lower median recurrence-free survival time than those without the XPF and XPC defect, and smoking can make this difference more remarkable. Our results suggest that XPF and XPC expression may be a potential predictive factor for bladder cancer, and smoking can not only influence the recurrence of bladder cancer as a single factor but also aggravate the results of the XPF defect and XPC defect.

## Introduction

Tumors of the bladder are the second most common cancers in the urologic field. Bladder cancer is the fourth most common cancer in men and ninth most common in women [1]. Of the patients, approximately 50% will experience a recurrence within 2 years after an initial diagnosis, and 16–25% will have recurrence after endoscopic resection [2]. Therefore, the frequent recurrence of bladder cancer is a major medical problem.

Tobacco smoking, as a predominant risk factor for bladder cancer, is responsible for about half the cases in men and a third in women [3], [4]. As tobacco-related carcinogens often form bulky DNA adducts, base damage, single-strand breaks and double-strand breaks [5]. In addition, because of the nature of the bladder as an important void organ, the urothelial cells are continuously exposed to many DNA-damaging reagents contained in the urine. Thus, DNA repair plays an important role in preventing deleterious DNA-damage-induced effects such as mutation accumulation and tumor occurrence [6].

There are several DNA repair pathways existing in human cells and each pathway effectively removes particular types of DNA damage [7]. Based on the type of DNA damage, the DNA repair pathways can be classified into nucleotide excision repair (NER), base excision repair, mismatch repair, and recombinational repair [8], [9]. The NER pathway is the major DNA repair pathway for repairing bulky DNA damage generated by most environmental factors to maintain genetic integrity and prevent the development of many disease [10], [11].

NER consists of approximately 30 proteins that remove helix-distorting lesions through four steps: (a) recognition of the DNA; (b) opening of a bubble around the lesion; (c) incision of the DNA upstream and downstream of the lesion by endonucleases; and (d) DNA resynthesis and ligation [12], [13]. XPA to G (xeroderma pigmentosum groups A–G), ERCC1 (excision repair cross complementation group1), RPA1, RPA2 (replication protein A1, replication protein A2) are the main proteins in this pathway and there are two damage recognition arms of the NER pathway: global genome repair (GGR) and transcription-coupled repair (TCR). GGR encompasses the noncoding parts of the genome, silent genes and the non-transcribed stand of active genes. TCR ensures that the transcribed strand of active genes is repaired with higher priority than the rest of the genome by using RNA polymerase II as a lesion sensor. Once the damage is recognized through one of these processes, the remainder of the repair process follows a convergent pathway [14], [15].

In this study, we investigated the mRNA expression of 9 genes (XPB to XPG, ERCC1, RPA1, RPA2)involved in the NER pathway in bladder cancer tissues with/without recurrence, compared with normal bladder cancer tissue. The patients without recurrence in 2 years were included in the non-relapse group and the patients with recurrence in 2 years served as the relapse group. Their tumor specimens resected in the first operation were studied. Then, the above genes with significantly different expression of mRNA were analyzed by an immunohistochemistry method (in 219 patients with bladder cancer) and the correlation between these genes and the recurrence of bladder cancer was determined.

Considering the tobacco smoking is a predominant risk factor for bladder cancer, we analyzed the clinical value of varied NER genes and smoking in 219 bladder cancers based on the clinical data by the Kaplan-Meier method and Cox proportional hazards regression.

## Materials and Methods

### 2.1 Ethics Statement

All the research protocol was approved by the ethics board of Bethune International Peace Hospital and XinQiao hospital at the Third Military Medical University and all participants provided written informed consent.

### 2.2 Study subjects

The study was designed with two independent sets.

The first set included 79 patients who underwent transurethral or partial cystectomy (mean age 62.4±12 years; 52 men and 27 women) in our department from 2007 to 2011. None of the 79 patients had received anticancer drugs before the first cystectomy. Among these 79 patients, 37 of them did not have a recurrence in at least 24 months after the first cystectomy and were still alive now, 29 of them had received a second cystectomy and 13 of them had died (5 cases received a second cystectomy and 8 cases did not). The tumor specimens of all surgical patients were collected and kept properly. The patients without recurrence in 2 years were included in the non-relapse group and the patients with recurrence in 2 years served as the relapse group. Their tumor specimens resected in the first operation were studied. The samples were snap frozen at the earliest possible time after resection.

The second set included 219 patients who were not the same as the 79 patients above. The paraffin wax-embedded tissues of bladder papillary urothelial carcinoma were collected from patients who underwent transurethral resection of bladder tumor in our department from 2002 to 2007. These tissues, according to the new WHO classification, were assigned pathologically to papillary urothelial neoplasm of low malignant (PUNLMP; 58 cases), low-grade papillary urothelial carcinoma (93 cases) and high-grade papillary carcinoma (68 cases). The patients without recurrence in 36 months were included in the non-relapse group and the patients with recurrence in 36 months served as the relapse group.The expression levels of NER genes in bladder cancer tissues of these patients were analyzed by immunohistochemistry method.

### 2.3 RNA extraction and cDNA synthesis

Tumour fragments were homogenised in RNA lysis buffer in ice with an Ultra-turrax and RNA was purified using the SV Total RNA Purification Kit. Retro-transcription to cDNA was done using the high capacity cDNA Archive Kit, in accordance with the manufacturer's suggestions.

### 2.4 Real-time PCR

Relative gene expression was measured in triplicate and normalized to *β*-actin(ΔCT)using TaqMan gene expression assays and the 7500 real-time PCR system for the following gene transcripts: XPB (Hs0554450_ml), XPC (Hs01104206_m1), XPD (Hs00361161), XPE(Hs00172018_ml), XPF(Hs00193342), XPG(Hs00164482), ERCC1(Hs01012161), RPA1(Hs00161419_m1), RPA2 (Hs00358315_m1). To ensure thatâ-actin itself did not change between different samples, the ratio ofâ-actin to a second reference gene GAPDH [16], was measured.

### 2.5 Immunohistochemistry

Tissue sections of 5 µm were deparaffinized, rehydrated in graded alcohols, and processed using the streptavidin immunoperoxidase method. In brief, sections were submitted to antigen retrieval by microwave oven treatment for 10 min in 0.01 mol/L of citrate buffer (PH 6.0). Slides were subsequently incubated in 10% normal serum for 30 min, followed by an overnight incubation at 4°C with the appropriately diluted primary antibody. The mice anti-human monoclonal antibody (XPC, XPF) was used at a 1∶100 dilution. After primary antibody, samples were incubated with biotinylated anti-mice or anti-rabbit immunoglobulins for 15 min at 37°C,followed by streptavidin peroxidase complexes for 15 min at 37°C. 3.3′-diaminobenzidine was used as the chromogen, and hematoxylin was used as a nuclear counterstain.

Immunohistochemical evaluation was conducted by at least two independent observers that scored the estimated percentage of tumor cells showing nuclear staining, independently of signal strength. Results were scored in urothelial carcinoma lesions by estimating the percentage of tumor cells showing characteristic nuclear staining. An arbitrarily defined 10% cutoff was taken to classify the TCC data into categorical groups (positive versus negative).

### 2.6 Statistical analysis

The statistical analysis was done by SPSS 13.0. Relative gene expression was calculated using 2^-ΔCT^, and unpaired two-tail t tests were used to identify significantly altered expressions in recurrent cancer tissues compared with non-recurrent cancer tissue. A contingency table was generated with the chi-square or Fisher exact probability test for immunohistochemistry data. Recurrence probability was calculated by the Kaplan-Meier method with statistical differences evaluated by the log rank test. The relative risk of recurrence from bladder cancer was estimated by a multivariate Cox proportional hazard model. For all statistical tests, p<0.05 was considered significant.

## Results

### 3.1 NER genes and the recurrence of bladder cancer

**Figure 1:**
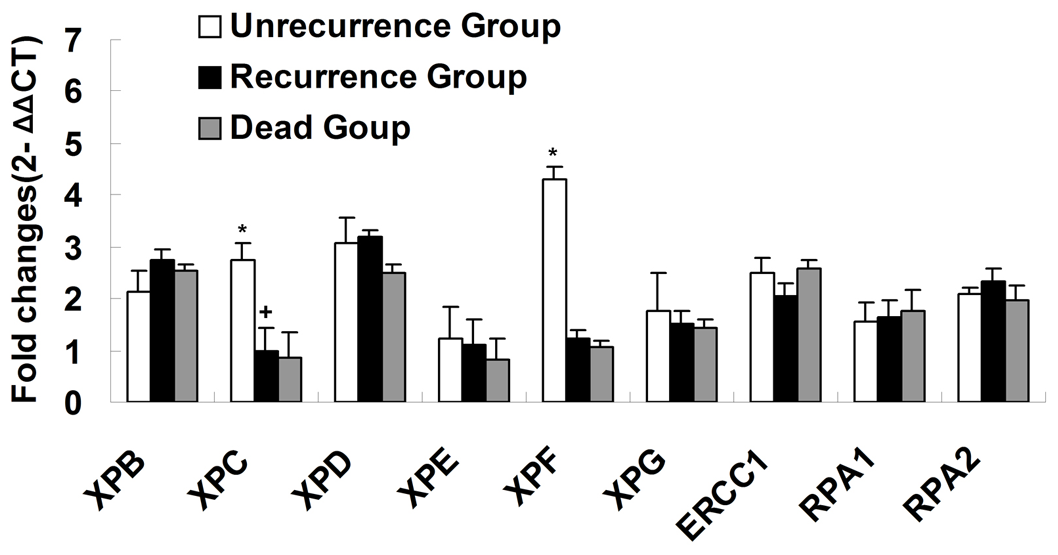
The RNA expressions of nine NER genes in bladder cancer tissues of three subgroups. NER genes transcript were detected by real-time quantitative RT-PCR in bladder cancers of three subgroups: Non-recurrence Group, patients who didn't have a recurrence in 2 years; Recurrence Group, patients who had a recurrence within 2 years; Dead Group, patients who were dead after the surgery within two years. The fold change was normalized against GAPDH and then all genes compared with the expressions of XPC in the Recurrence Group(+signal). *, P<0.01 compared with the.Non-recurrence Group.

To clarify the correlation between NER mechanism and bladder cancer relapse, the expression levels of NER genes including XPB to XPG, ERCC1, RPA1 and RPA2 in cancer tissues from 79 patients with bladder cancer were determined by real-time PCR. In these 79 patients, 37 patients had no relapse in 24 months, and these were included in the non-relapse group; 29 patients had a relapse in 24 months and were included in the relapse group; and the other 13 patients died and were included in the death group. The study findings showed the expression levels of XPC and XPF were significantly reduced in the recurrence group and death group compared with the non-relapse group (Fig. 1 and Table 1). However, XPB, XPD, XPE, XPG, ERCC1, RPA1 and RPA2 did not change significantly. Compared with the recurrence group, the expression levels of XPC and XPF were far lower in the death group; however, it had no statistic significance.

**Table 1:** Decreased XPF and XPC expression in 79 patients of human bladder cancer.

	XPF(-)	XPC(-)	Total tumors
**Grade**			
**Papillary neoplasm of malignant potential**	8(42.1%)	7(36.8%)	19
**Low-grade papillary carcinoma**	17(48.6%)	14(40.0%)	35
**High-grade papillary carcinoma**	15(60.0%)	12(48.0%)	25
**P value**	P^a^>0.05	P^a^<0.05	
	P^b^<0.01	P^b^<0.05	
	P^c^>0.05	P^c^>0.05	
**Prognosis status(Within 2 years)**			
**Non-relapse group**	16(43.2%)	14(37.8%)	37
**Relapse group**	17(58.6%)	15(51.7%)	29
**Dead group**	7(53.8%)	5(38.5%)	13

### 3.2 The expression levels of XPF and XPC and the pathology of bladder cancer

**Figure 2:**
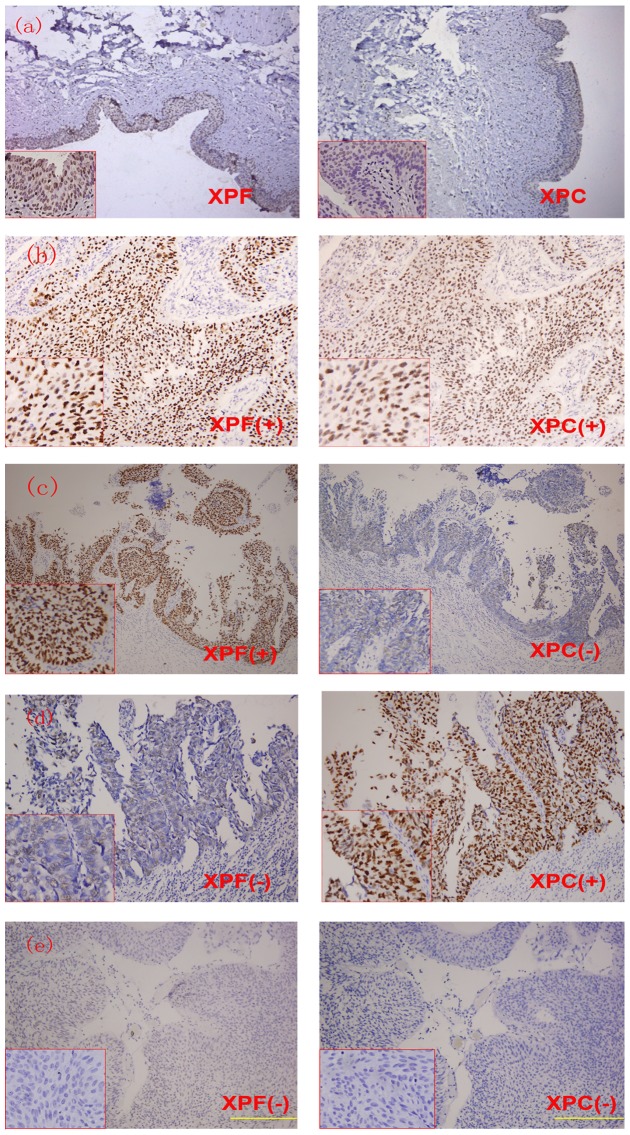
Immunohistochemical staining of paraffin embedded bladder urothelial carcinoma. (a)The expressions of XPF and XPA in normal bladder tissue. (b) Positive XPF expression and positive XPC expression. (c) Positive XPF expression and negative XPC expression. (d) Negative XPF expression and positive XPC expression. (e) Negative XPF expression and negative XPC expression.

To investigate the relationship between XPF, XPC and the development of bladder cancer, the connection between the expression levels of XPF and XPC and the pathology of bladder cancer was analyzed. It was found that the proportions of XPF (−) and XPC (−) were 54.4% and 50.0% in the high grade papillary cancer tissue, 39.7% and 37.9% in PUNLMP cancer tissues and 49.5% and 45.2% in low grade papillary cancer tissues (Table 2 and Fig. 2). The results of statistical analysis on immunohistochemistry data suggested that the proportions of XPF (−) and XPC (−) were significantly higher in high grade papillary cancer tissues than in PUNLMP cancer tissues (P<0.05). No statistical difference was observed between the PUNLMP and low-grade tumors (P>0.05). Furthermore, the proportions of XPF and XPC with concurrently reduced expression were significantly higher in high grade papillary cancer tissues than in PUNLMP cancer tissues (P<0.05).

**Table 2:** The associations of attenuated XPF/XPC expression with tumor grade and the patients' smoking status in 219 patients with bladder cancer.

	XPF(−)	XPC(−)	XPF(−)XPC(−)	Total tumors
**Grade**				
**Papillary neoplasm of malignant potential**	23(39.7%)	22(37.9%)	9(15.5%)	58
**Low-grade papillary carcinoma**	46(49.5%)	42(45.2%)	24(25.8%)	93
**High-grade papillary carcinoma**	37(54.4%)	34(50.0%)	19(27.9%)	68
**P value**	P^a^<0.01	P^a^<0.01	P^a^<0.01	
	P^b^<0.01	P^b^<0.01	P^b^<0.01	
	P^c^>0.05	P^c^>0.05	P^c^<0.05	
**Smoking status(Pack-years)**				
**never smoked**	31(79.5%)	29(74.4%)	12(30.8%)	39
**<20 Pack-years**	28(59.6%)	20(42.6%)	13(27.7%)	47
**20-50 Pack-years**	21(30.9%)	25(36.8%)	12(17.6%)	68
**>50 Pack-years**	25(38.5%)	24(36.9%)	15(23.1%)	65

Note P^a^ value is the comparison between the group of low-grade papillary carcinoma and the group of papillary neoplasm of low malignant potential; P^b^ value is the comparison between the group of high-grade papillary carcinoma and the group of papillary neoplasm of low malignant potential; P^c^ value is the comparison between the group of high-grade papillary carcinoma and the group of low-grade papillary carcinoma(Table 1 and Table 2).

### 3.3 Expression of NER genes and smoking

**Figure 3:**
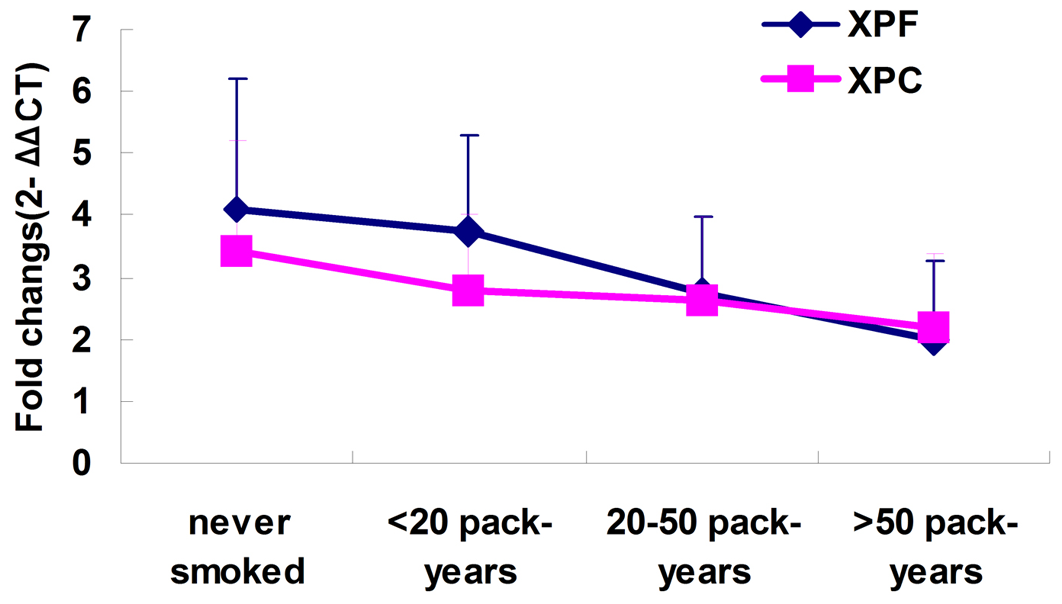
Analysis of relative XPF and XPC mRNA levels in bladder cancer tissue with different smoking status. The expressions of XPF and XPC in 79 the bladder cancer of 4 smoking subgroups. The coarse solid line represents the mean CT value and the error bars represents the standard error.

The connection between the expression levels of NER genes and smoking in 79 bladder cancer patients was analyzed. The smoking situation was divided into 4 levels: never smoked, <20 pack-years, 20–50 pack-years and>50 pack-years. The study findings showed that although no statistic significance existed among the 4 smoking groups, there was still a definite trend. The expression levels of XPF and XPC were slightly lower in the tumor tissues of heavy smokers than in those of mildly smoking patients (Fig. 3).

To further explore the connection between the expression of NER genes and smoking, the expression levels of XPF and XPC in 219 bladder cancer patients and their smoking situations were analyzed. We found no difference in the proportions of XPF (−) and XPC (−) among the <20 pack-years group, 20–50 pack-years group and never smoked group. Interestingly, the proportions of XPF (−) and XPC (−) in the>50 pack-years group were higher than those in the never smoked group and <20 pack-years group (Table 2 and Table 3).

**Table 3:** Univariate analysis of clinical characteristic influence on recurrence in 219 patients with bladder cancer.

Parameter	No.patients	Median recurrence months	P Value (log rank test)
**Gender:**			0.407
**Women**	51	23.90	
** Men**	168	25.63	
**Age:**			0.495
** 60 or less**	99	25.20	
** Greater than 60**	120	25.25	
**Pathological grade:**			0.037
** PUNLMP**	58	27.60	
**Low grade papillary Ca**	93	25.18	
** High grade papillary Ca**	68	23.25	
**Pathological stage:**			0.000
** Ta-T1**	137	27.99	
** T2-T4**	82	22.62	
**XPC:**			0.003
** Positive**	121	27.60	
** Negative**	98	22.30	
**XPF:**			0.001
** Positive**	106	27.56	
** Negative**	113	22.74	
**Smoking status**			0.037
** never smoked**	39	27.94	
** <20 pack-years**	47	24.73	
** 20–50 pack-years**	68	26.06	
** >50 pack-years**	65	21.76	

### 3.4 Expression of XPF and XPC bladder cancer recurrence

**Figure 4:**
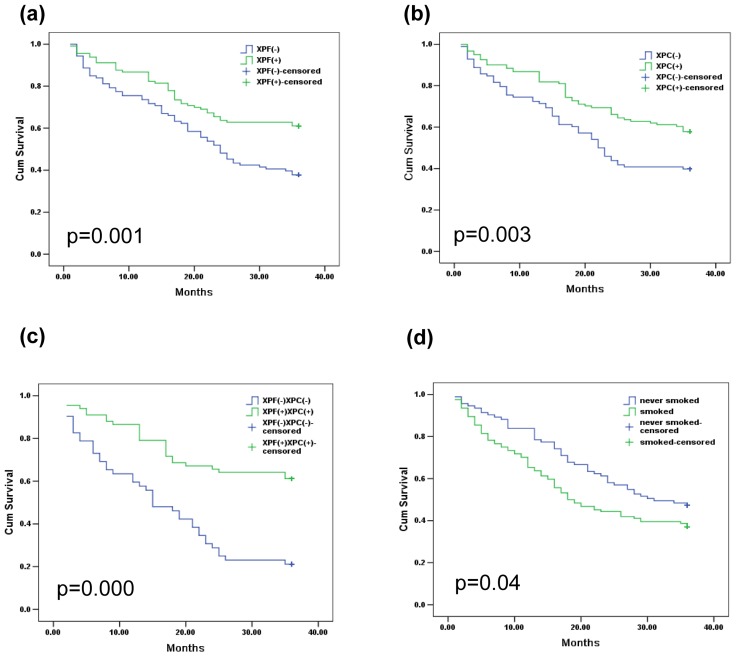
Expression of XPF and XPC bladder cancer recurrence. Kaplan-Meier Curves using log rank tests for the recurrence of bladder cancer in patients. (a) The recurrence of bladder cancer in patients with XPF(−) and XPF(+); (b) The recurrence of bladder cancer in patients with XPC(−) and XPC(+); (c) The recurrence of bladder cancer in patients with XPF (−) combined with XPC(−) and XPF(+) combined with XPC(+), simultaneity; (d) The recurrence of bladder cancer in patients with never smoked and ever smoked. *Cum, cumulative*.

To further investigate the relationship between the reduced expression levels of XPF and XPC and the development of bladder cancer, the connection between the expression levels of XPF and XPC with bladder cancer relapse was analyzed. Fig. 4 shows the Kaplan-Meier curves of the two genes and smoking status with the adjusted P values. We found that the patients with low level of XPF and XPC had a higher recurrence rate than those with high level of XPF and XPC (Fig. 4a and 4b P<0.05), moreover, this difference was more obvious in the group XPF(−)XPC(−) (Fig. 4c). Among the clinicopathologic variables, tumor grade and stage resulted as significant prognostic factors at univariate analysis (Table 3). The multivariate analysis by Cox was performed, including all the variables significantly associated with survival at the univariate and indicated that the stage of disease was the only independent factor predicting patients' outcome (Table 4).

**Table 4:** Cox multivariate regression analysis of potential recurrence predictive factors in patients with bladder cancer.

Variable	Category	RR(95% CI)	P Value
**Age**	Less than 60/60 or greater	1.265(0.857–1.869)	0.237
**Grender**	Men/Women	0.794(0.510–1.236)	0.307
**Grade**	High/Low grade papillary Ca/PUNLMP	1.686(0.599–4.024)	0.001
**Stage**	T2-3/Ta-1	1.944(1.302–2.902)	0.001
**XPC**	(−)/(+)	1.694(1.147–2.502)	0.008
**XPF**	(−)/(+)	1.652(1.118–2.441)	0.012
**Smoke status**	never smoked/>50 pack-years	0.684(0.310–1.540)	0.043

### 3.5 The smoking status and bladder cancer recurrence

**Figure 5:**
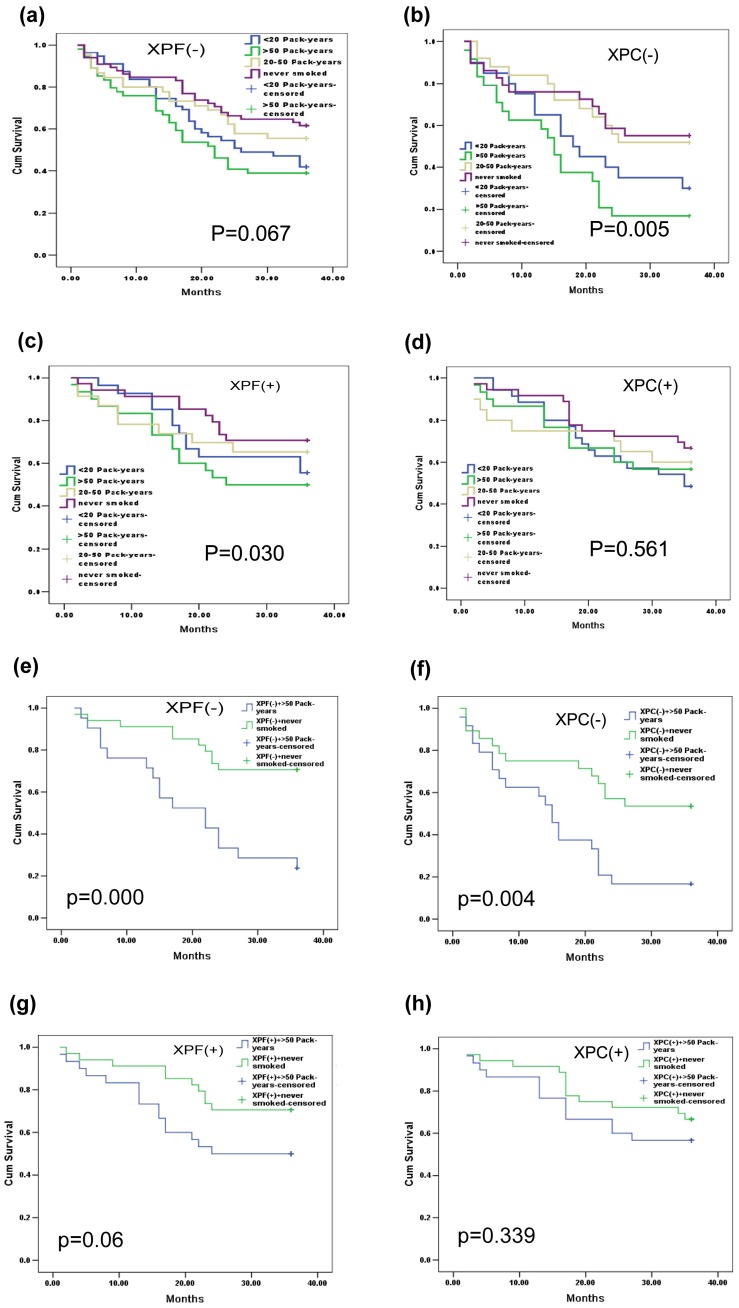
The smoking status and bladder cancer recurrence. Kaplan-Meier Curves using log rank tests for the recurrence of bladder cancer in patients. (a) The recurrence of bladder cancer in XPF(−) group with different status of smoking (never smoked, <20 pack-years, 20-50 pack-years and>50 pack-years); (b) The recurrence of bladder cancer in XPC(−) group with different status of smoking; (c) The recurrence of bladder cancer in XPF(+) group with different status of smoking; (d) The recurrence of bladder cancer in XPC(+) group with different status of smoking; (e) The recurrence of bladder cancer in patients of XPF(−) with never smoked and XPF(−) with smoked more than 50 pack-years;(f) The recurrence of bladder cancer in patients of XPC(−) with never smoked and XPC(−) with smoked more than 50 pack-years;(g) The recurrence of bladder cancer in patients of XPF(+) with never smoked and XPF(+) with smoked more than 50 pack-years; (h) The recurrence of bladder cancer in patients of XPC(+) with never smoked and XPC(+) with smoked more than 50 pack-years.

It had been confirmed that smoking is associated with bladder cancer [30], and we found that smoking was also associated with the recurrence of bladder cancer. Fig. 4d shows the Kaplan-Meier curves of smoking status with the adjusted P values, the patients who had a history of smoking had a higher recurrence rate than the patients who didn't have the smoking history. Moreover, we found that the smoking status was not a separate factor to bladder cancer, it can work with XPF and XPC genes simultaneously, especially in the patients with a low level of XPF (Fig. 5 a and c) and XPC (Fig. 5 b and d). In addition, the amount of smoking of the patient was related to the recurrence of bladder cancer (Fig. 5), especially in the group of XPF(−) and XPC(−) (Fig. 5 e and f).

## Discussion

Bladder cancer relapse is a clinically common problem. In this study, we investigated the connection between bladder cancer relapse and NER genes. The study results suggested that two NER genes, XPF and XPC, were involved in the relapse of bladder cancer. Moreover, we also found that as an external factor, smoking was not only involved in the relapse of bladder cancer independently, but also combined with NER genes to play a malignant role.

XPF,also known as the excision repair crosscomplementing group 4 (ERCC4), is critically involved in the NER pathway [17]. XPF hasan important role in recombination repair, mismatch repair and possibly immunoglobulin class switching [18], [19]. XPF contains the catalytic domain of the nuclease and ERCC1 is required for DNA binding and stabilization of XPF. The ERCC1-XPF complex could remove 3′ single-standed flaps from DNA ends and cleaves the 5′ side of a bubble in NER to excise toward the lesion [20]. We found XPF was significantly less in tumor tissues of relapsed bladder cancer and the results from pathology comparison showed that the possibility of relapse within 3 years was higher for bladder cancer patients with XPF (−) than for those with XPF (+). It indicated clearly that XPF was involved in the relapse of bladder cancer, which was consistent with the results from Z Zhang et al [21]. They observed that the polymorphism of the XPF-357 site was closely correlated with the expression of XPF and further affected the relapse of bladder cancer. However, they had not investigated the different expression levels of XPF in tumor tissues of relapsed and non-relapsed bladder cancer and also had not derived a clear conclusion. We not only investigated the different expression levels of XPF in tumor tissues of relapsed and non-relapsed bladder cancer, but also studied the connection between the proportion of XPF (−) and bladder pathological grading. We found the proportion of XPF (−) was significantly higher in high grade papillary cancer tissues than in PUNLMP cancer tissues and low grade papillary cancer tissues, suggesting XPF played not only an important role in the relapse of bladder cancer but also a protective role in the development of bladder cancer. The reduced XPF was involved in the development of bladder cancer.

XPC is a DNA damage recognition protein that plays an important role in the NER process. The XPC protein binds tightly with an HR23B protein to form a stable XPC-HR23B complex. Studies indicate that the XPC-HR23B complex is the first protein component that recognizes and binds to the damaged sites [22]. The XPC protein might also play an important role in other DNA damage-induced cellular responses, including cell cycle checkpoint regulation and apoptosis [23]–[25]. XPC defects have been found in many types of cancer, including lung and skin cancer [26], [27]. XPC knock-out transgenic mice studies reveal the high incidence of a predisposition to many types of cancer [28]. Although several studies have demonstrated the close relationship between XPC and the development of bladder cancer, there is still no evidence that XPC is involved in the relapse of bladder cancer.

We found XPC was Attenuated expressed in relapsed bladder cancer than non-relapsed bladder cancer. It suggests that the reduced XPC may be involved in the relapse of bladder cancer. To confirm this hypothesis, we analyzed the connection between the expression levels of XPC in tumor tissues of 219 bladder cancer patients and their prognosis and found XPC (−) patients had a significantly higher relapse rate. Moreover, the proportion of XPC (−) was correlated with the pathological grading of bladder cancer. This is consistent with the results from Chen et al: XPC defeat was closely related with and involved in the development of bladder cancer [29].

Although the low expression of XPF and XPC was involved in the relapse of bladder cancer, the comprehensive analysis found that there was no synergic enhancement effect between the reduced expression of XPF and XPC. The case-controlled analysis showed that the relapse rate of the patients with concurrently reduced expression of XPF and XPC was not significantly higher than that of the patients with only reduced expression of XPF or XPC. This suggests that XPF and XPC are both related with the relapse of bladder cancer and involved in several cellular response pathways. However, as for the replace of bladder cancer, XPF and XPC play their roles in the same mechanism, the NER mechanism, but not in different mechanisms.

Many studies have showed that smoking plays a malignant role in the development of bladder cancer [30]–[32]. Our study further confirmed this conclusion. By carefully analyzing the smoking status, the expression of NER genes and the relapse of bladder cancer, we have derived the conclusion that smoking is not only involved in the relapse of bladder cancer independently, but also combines with NER genes like XPF and XPC to play a malignant role simultaneously, which further aggravated the malignant outcomes of bladder cancer. Previous studies suggested that smoking might affect the relapse of bladder cancer through the NER mechanism. However, our study showed smoking not only affected the protective role of the NER mechanism in bladder cancer, but also probably influenced other protective mechanisms during the development of bladder cancer.

Despite our study showing that XPF, XPC and smoking were closely related with the relapse of bladder cancer, the reason for low expression of XPF and XPC was still unclear. In addition to the polymorphism of XPF and XPC promoters may affect the expression of these genes, probably there are some unknown mechanisms affecting the transcription and translation of XPF and XPC genes, even affecting their protein degradation and protein efficiency, etc. To thoroughly elucidate the biological mechanism of bladder cancer relapse, these fields need further investigation. However, the fact that XPF and XPC are involved in the relapse of bladder cancer brings a new reliable clue for further studies on the mechanism of bladder cancer relapse.
